# The Effect of Loading on the Diffusivity of Chlorides in Mortar

**DOI:** 10.3390/ma12162527

**Published:** 2019-08-08

**Authors:** Marta Cabeza, Belén Díaz, X. Ramón Nóvoa, Carmen Pérez, M. Consuelo Pérez

**Affiliations:** ENCOMAT group, University of Vigo, EEI, Campus Universitario, 36310 Vigo, Spain

**Keywords:** chloride diffusion coefficient, impedance spectroscopy, electrical resistance, compressive load

## Abstract

This study focuses on the effect generated by a compressive load, in the range 15%–60% of the ultimate load (F_u_), in the chloride penetration rate of cement-based materials. The modifications produced in the microstructure influence the transport properties, and, thus, the validation of several interesting parameters, such as, the load value and the loading time, including both static and dynamic loading modes, was evaluated. This analysis was performed by impedance spectroscopy (IS), a non-destructive technique that allowed, after the appropriate modeling analysis, the assessment of the resistivity of the sample, a parameter that has been correlated to the diffusion coefficient in a previous investigation. The experimental arrangement was designed to allow the recording of the impedance spectra under the effect of a compressive load and, thus, the real-time monitoring of the chloride diffusivity was provided. An increase in the diffusion coefficient was verified for a load at 60%F_u_ whereas no variations were obtained for the load fixed at 30%. A relevant difference could be checked if the values were measured once the load was removed, showing the importance of the precise loading stage for the chloride diffusion study.

## 1. Introduction

A review by Yao el al. has summarized the importance of the combined action of both mechanical and environmental factors to assess the durability of concrete structures [1]. The review points, in general, to an accelerated mechanical degradation as well as to an increase in the aggressive species penetration. As a result, the service life of the reinforced concrete structures could be reduced. For the particular situation of the chloride penetration ability under the influence of a service load, there is no generalized conclusion on the cooperative effect produced by both factors. 

Concerning the possible consequence of these two variables on a cement-based material, two opposite concepts are referred in the literature: One about pore compaction, giving a reduced diffusivity [2,3,4], and the other about crack formation and/or development, making the contrary result [5,6,7,8]. The diverse tested stress levels along with the different methodologies followed in these investigations, in both the experimental setup and the chloride diffusivity assessment, seem to be the reason for such a discrepancy in the results. 

No standardized procedure has been so far defined to report the loading effect and then there is no agreement concerning the critical stress to produce an irreversible damage [9]. On the other hand, most of the reviewed studies compile the results obtained with different experimental arrangements and most of them do not take into account the chloride permeation rate under a compressive load but it is assessed after the load has been removed [5,10,11,12,13,14]. Thus, these reports are not conclusive on the real stress condition, and they do not provide the direct correlation between the external stress and the diffusion rate.

A realistic approach was provided by Yu et al. [15], who recorded the reinforcement corrosion rate under an aggressive environment along with a flexural stress. A reduction in the time required for the corrosion initiation was proved but the arduous methodology used makes almost impossible the transference to the real structure monitoring. On the contrary, other authors pointed to a reduced chloride penetration depth obtained under a long-term compressive force [16]. A clear correlation between the mechanical action and the chloride diffusion has not been established so far and, thus, research is still required to clarify the combined effect of both variables. 

Apart from these studies, Basheer et al. have performed an interesting study with an innovative testing arrangement where the real influence of several service loads could be tested [8]. They have tested several concrete mixtures and they found an increased chloride diffusivity above a certain stress value, in the range of 50% of the ultimate stress value, pointing to the formation of microcracks as responsible for such a detrimental variation. As a general comment on the reliability of this study, one could state that the diffusivity could be influenced by the loading time, a variable that is not considered here since it was defined by the own migration test. On the other hand, the results of the migration tests could be inaccurate due to the influence of the voltage employed to perform the experiment in the microstructure of cementitious materials [17,18]. 

These two drawbacks, along with a previous study carried out in our research group [19], confirm the motivation of the current investigation. In our previous study, we evidenced by impedance spectroscopy (IS) the modifications induced in the microstructure of cement paste specimens by small compressive loads. 

Moreover, a simple procedure based on IS to determine the chloride diffusion coefficient was also designed previously [17]. Here the apparent resistivity of the cement-based materials was numerically correlated to the chloride diffusivity. Thus, it seems conceivable to detect the real-time influence of a compressive load on the chloride diffusion provided that changes in the resistivity could be recorded. Actually, Chung el al. have revealed changes in the resistivity under static and dynamic loads, encouraging results that have also motivated this study [20,21,22] Therefore, a specific experimental arrangement, later described, was designed for the purpose of this research that allowed the validation of the resistivity from impedance measurements under a sustained load. Then, the real-time monitoring of the chloride diffusion was assessed. 

## 2. Materials and Methods 

Mortar samples were prepared following the procedure described in the EN 196-1 standard. Portland cement CEM I 42.5 R and standardized sand (EN 196-1) were used. The fresh mixture, composed of 1350 g of sand, 450 g of cement, and 180 g of water, was poured in metallic molds with three separated compartments so that 4 × 4 × 16 cm^3^ prims were obtained. Flexural and compressive tests were previously performed in order to characterize the as-prepared samples according to the EN 196-1 standard. The average data corresponding to the ultimate loads are presented in Table 1. The mercury intrusion porosimetry (MIP) test was also performed, as described elsewhere [23,24]. The main results from MIP, porosity and average pore size, are presented in Table 1 as well. 

**Figure 1:**
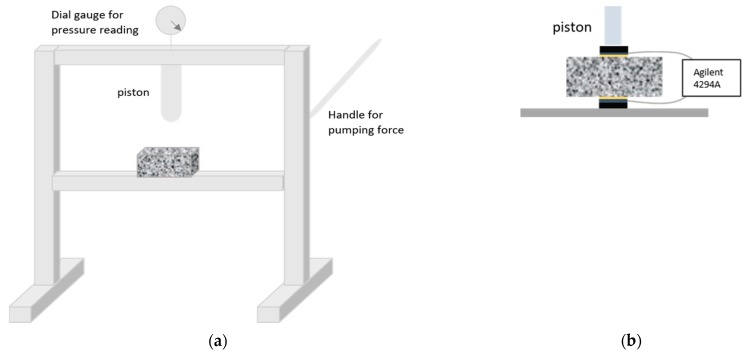
Experimental arrangement used to register the impedance spectra under a sustained load: (**a**) Hydraulic press and sample positioning and (**b**) sample and electrode configuration for the impedance analysis.

A hydraulic press (EBERTH, Surheim, Germany) was used to apply the load and the experimental setup was fixed as illustrated in Figure 1. Figure 1a shows a sketch of the press along with the sample positioning; whereas Figure 1b details a closer view of the sample and electrodes arrangement for the impedance measurements, adapted from the original design by Gu et al. [25]. 

Graphite electrodes were located at both sides of the samples to perform the impedance measurements in line with the load direction. Damp sponges were used between the graphite and the sample to optimize the ionic contact. Two rigid polymeric pieces (~1 mm thick) were placed, above and below the sample, to help with the electrode fixing and to define the loading area (40 × 40 mm^2^, equivalent to the size of the graphite electrodes and the sponges). Other configurations, with the measuring electrodes placed perpendicular to the piston, were tested but some problems with the electrode fixing arose and they were finally discarded.

The press must be located as near as possible to the impedance analyzer (Agilent, Santa Clara, United States) in order to reduce the inductive response of the cables to the whole measurement. In any case, the impedance response of the cables, along with the pressed electrodes and sponges, was mathematically removed from the whole impedance to get a more precise information from the sample. An Agilent 4294A was used for the measurements, able to sweep the frequency range from 40 Hz to 110 MHz. The voltage amplitude was fixed to 50 mV and nine points per decade were recorded. 

The mortar samples must be conditioned prior to the loading tests in order to guarantee a chloride concentrated solution inside the pores, a requisite for the diffusion coefficient validation, as defined elsewhere [17]. Here, one of the parameters obtained from the impedance fitting, in particular that concerning the sample resistance named *R*_1_, provides the coefficient assessment according to Equation (1):

(1) $$D_{Cl^{-}}=\frac{A}{R_{1}}$$

where *A* is a constant value depending on the chloride concentration (*C_Cl_*^−^) and the sample geometry (thickness, *d*, and cross-section area, *S*) as indicated in Equation (2):

(2) $$A=\frac{R*T*d}{S*1.639*F^{2}*C_{Cl^{-}}}.$$

This is a non-destructive procedure that allows the diffusivity calculation in the same device where the load is being applied, with no need of transferring the sample to additional specific devices. Then it gives real time information on the influence of the load on diffusivity.

Three different experiments were performed in order to understand the influence of the load: The load value, the loading time, and the dynamic loading. The experimental details are summarized in Table 2.

The first tests were conducted in order to define the influence of an isolated force applied for a short period (~10 s, time required for the impedance measurement). The applied loads were increased from 9.8 to 39.2 kN, which correspond to values between 15% and 60% of the ultimate load (F_u_). The impedance measurements were performed just after the selected load had been achieved.

In another group of tests, several samples were subjected to fixed load values, 19.6 kN (30% of the F_u_) and 39.2 kN (60% of the F_u_), for a period of 30 min. This maximum testing period was fixed in order to avoid the drying of the samples, which may influence the ionic conduction. The impedance data were recorded just after the load was applied and, after that, one measurement each 5 min was performed. After this period, the load was released and the impedance measurements were still recorded for 15 min. This second part allows gathering information about the ability of the specimens to recover the original microstructural condition.

The last group of experiments were focused on determining the microstructural modifications under a cyclic load. Each cycle, which was repeated 10 times, consisted of a loading step of 39.2 kN for 1 min (with the impedance measurement after that period) and a second unloading step for 4 min. 

## 3. Results and Discussion

### 3.1. Effect of an Isolated Force

**Figure 2:**
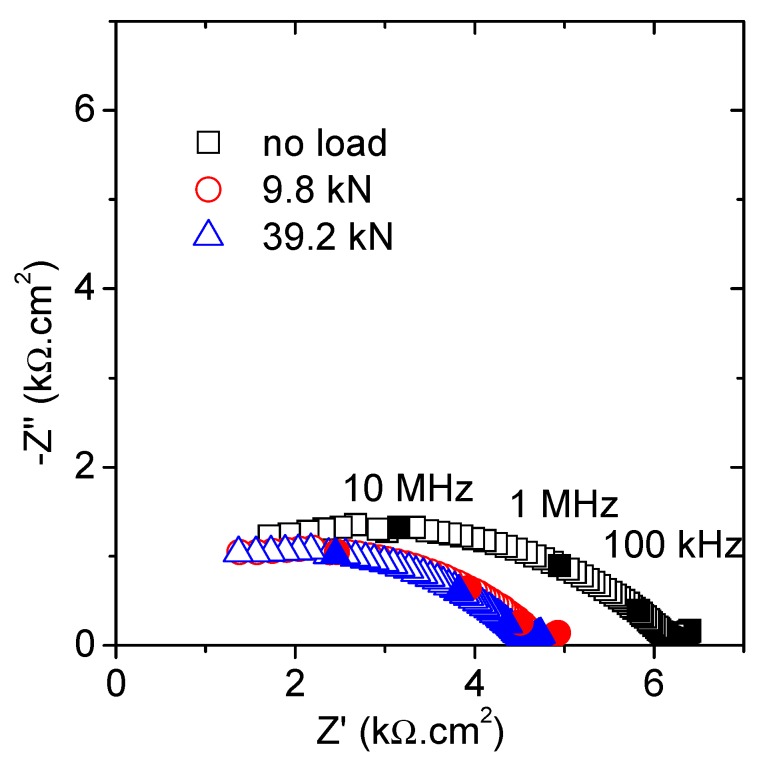
Nyquist plots obtained under several constant compressive forces.

Firstly, the influence of a particular loading state will be evaluated. Thus, Figure 2 shows the Nyquist plot including the results obtained for the unloaded, the lowest, and the highest loading states. The obtained graphs correspond well with the expected results for such an impedance with contacting setup. The high-frequency semicircle (at f > 10 kHz) describes the specimen microstructure and the short tail (for f < 10 kHz) derives from the interfacial electrode polarization response [26,27]. This interferencial response was not taken into account in any further analysis. A significant decrease in the high frequency arc diameter was observed once the lowest load (9.8 kN) was applied, but no major changes were revealed after the subsequent increments up to 39.2 kN.

**Figure 3:**
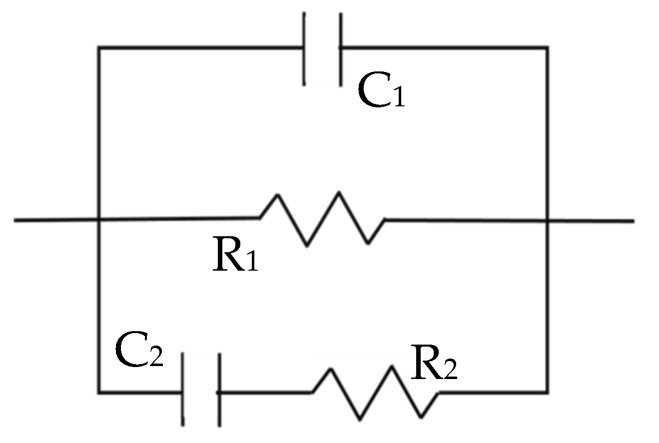
Equivalent circuit employed for the data fitting [17,28,29].

The fitting of these results was completed using the equivalent model, presented in Figure 3, that has been already validated for these types of samples in previous publications [17,28,29]. The parameters have been related to the properties of the material as follows: Capacitance, C_1_, refers to the solid phase dielectric properties, R_1_ is correlated to the ionic conduction through the percolating pores, and corresponds to the high-frequency arc diameter, and the second time constant defined by R_2_ and C_2_ measures the response of the ions located inside the isolated pores. 

The best fitting values are presented in Table 3. The most significant variations, according to the graphical appearance, were linked to the resistance of the specimen (R_1_). A sharp drop, ~23%, was measured after application of the lowest load, but almost no reduction was obtained when increasing the load. This variation was significantly greater than that expected due to the strain generated by the compressive load. In the same line as the studies by Gu et al. [25,30], the formation of microcracks and further filling with the electrolyte could explain such a reduction in the semicircle diameter. Then, new conduction paths, parallel to those already existent, are created when loading, even for load levels around 15% of the ultimate force. It seems that the load value, at least in the analyzed load range, does not play a significant role with respect to the mortar damage. As expected, no important variations were obtained in the dielectric capacitance (C_1_), in agreement with previous results [19]. 

The parameters of the second time constant exhibit some variation, again more relevant after the first (lowest) load was applied. The reduced resistance and increased capacitance suggest the formation of more non-percolating cavities, equivalent to those above-referred microcracks. The size of these pores lies in the expected range for the as-prepared mortar sample (Table 1), around 20 nm for all the tested conditions. This value was obtained following the connection between the time constant ($\tau_{2}=R_{2}\times C_{2}$) and the pore size (δ) through the diffusion coefficient of the electrolyte species (D): $\delta =\sqrt{\tau_{2}\times D}$ [5,12]. The data given by MIP and compiled in Table 1 are in good agreement to these values. No differences are revealed in terms of the pore sizes, at least in the covered load range up to 60%F_u_. 

### 3.2. Load Sustained for a Longer Period

**Figure 4:**
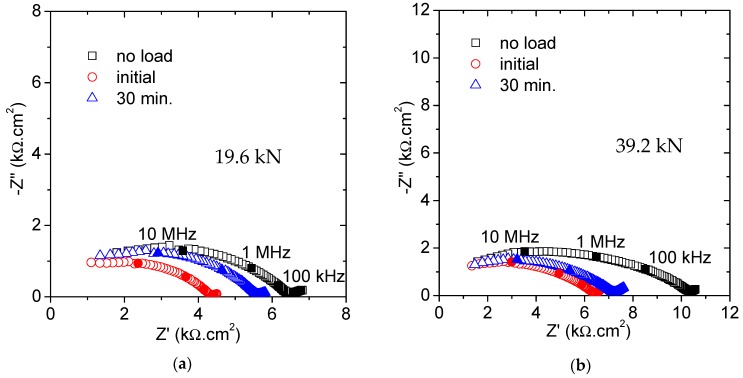
Nyquist plots obtained for 30 min loading: (**a**) At 19.6 kN (30%F_u_) and (**b**) at 39.2 kN (60%F_u_).

In case the mortar specimens are subjected to a fixed load for a longer period, some differences are detected according to the force level applied. Figure 4 shows the Nyquist plots obtained for 19.6 and 39.2 kN applied for 30 min. It is worth mentioning that the original values (named no load), in spite of being taken from the same mixture, are slightly different for Figure 4a,b. This fact is no way reflecting any inaccuracy in the measurements but it likely shows problems of segregation among the several prepared prisms. 

A sharp decrease in the impedance response was measured once the load was applied in both examples. After this initial variation, the trend changed and an increment in the resistance was obtained, more important for the case of the lowest load. The initial decrease agrees with the previous observations and the formation of microcracks is again proposed. Loading for a longer period seems to produce the opposite effect, so that some capillary pores are blocked and their ability for ionic conduction is reduced. This observation, although in a slightly different context, was also observed in the previous investigation by Gu et al., where a densification of the porous matrix was suggested [25,30]. The reduction in the pore connectivity has also been suggested by Wang et al. who studied the chloride diffusivity under a small compressive force [16]. Then, two opposite factors must be considered when the mortar specimens are under the influence of a compressive force for some time: New conduction paths are created, and some paths are then obstructed.

The coexistence of concrete damage and densification has been also proposed by Han et al. for stresses below 70% of the compressive strength [3]. According to the data obtained along the 30 min of loading, the reparation process seems to exceed the damage effect. Thus, the microcracks developed under the loading conditions considered in this study remained stable, i.e., once their final length was reached no further propagation was produced. The development of unstable cracks, giving the failure of the structure, would be expected above 75%F_u_ [31].

For the specimen under the highest force, the whole structural damage was more intense, with the last recorded impedance value significantly smaller than the original one. The impedance obtained after 30 min under 9.6 kN was very near to the original unloaded graph. It seems that below a certain stress value no great microstructural modifications would be expected during the loading period. Since the experiments were conducted under these two specific load states, that critical value cannot be precisely defined but would be in the range between 30% and 60% of the ultimate load. 

The fitting results are compared in Table 4. The major change refers again to the percolating resistance, R_1_. The other parameters are helpful to validate the modeling analysis. Both the capacitance, C_1_, and the non-percolating pores sizes (δ) are in the expected range, with no significant modifications throughout the experiments.

**Figure 5:**
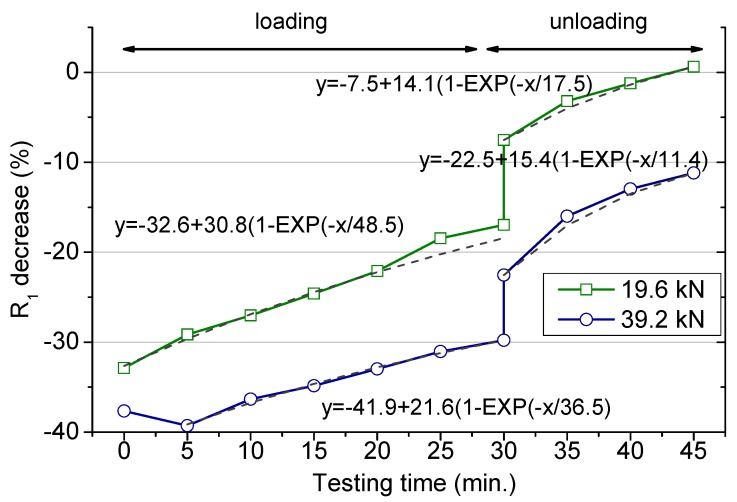
Percentage decrease of the percolating resistance (R_1_ in the model presented in Figure 3) during the loading period (up to 30 min) and after the load removal (after 30 min).

The full variation of the R_1_ values along the loading period are presented in Figure 5. The second part after releasing the load (after 30 min) will be separately discussed. The values are presented as percentage decrease in comparison to the original value (no load) to facilitate the comparison. For the smaller force, the sharp initial drop, about 35%, was followed by a continuous recovering. For the highest load, essentially the same, with the only difference that the smaller resistance was measured after 5 min once the test started. As above indicated, the partial recovery of the resistance along the loading test was more remarkable for the smaller load. The specimens drying would produce a resistance increment that would be equivalent independent of the loading state. According to the differences obtained for the two loading conditions, its influence, if any, is masked by the own loading effect.

The two structural changes already mentioned are valid to explain the variations recorded in the resistance. In any of the studied circumstances, R_1_ values tend to recover after the initial decrease, and the higher the load applied the higher the R_1_ decrease percentage at any loading stage. Moreover, for the case of the highest load, a slower resistance recovery was obtained. This observation agrees with definitive modifications along the whole loading period that the material is not able to heal. The time-dependent strain under a compressive sustained load, known as the creep phenomenon [32], would be expected to produce the opposite variation in the resistance. This deformation, if any, remains masked under the whole resistance variation, making this effect less relevant in terms of the mortar conductivity according to the obtained results. The short-time loading test may be not enough to identify this phenomenon. 

In order to extrapolate these results to longer loading times, the changes of the R_1_ were fitted to exponential functions. The obtained equations are included in Figure 5. According to the fits, the R_1_ percentage decrease reached stationary values, −1.8% and −20.3%, under 19.6 and 39.2 kN, respectively. The characteristic time for these evolutions was shorter (36.5 min) for the higher load than for the smaller one (48.5 min), which points to more rapid microstructural modifications at 39.2 kN load. Thus, no major changes would be expected in the resistance for a long period under a load in the range of the 30% of the ultimate strength. A higher load definitely produces a permanent damage at the microstructural level, particularly defined as an increased in the percolating pores connectivity. 

The impedance measurements were still recorded for some time after the load was released. The R_1_ values slightly increase after a sudden rise as shown in Figure 5. This variation agrees with the theory of the creep recovery, when a load is removed, the strain drops instantaneously with a gradual decrease after that [32]. Such a variation produces a sharp rise in the electrical resistance followed by a continuous increase, as reproduced in Figure 5. 

These data obtained after releasing the load have been again successfully fitted to exponential functions. The fits were obtained assuming the beginning of the unloading stage as time zero. The fit allows obtaining the end percent decrease in the resistance, although this information will be just valid under the specific experimental parameters (load value and loading time) followed in this study. Thus, after removing a load corresponding to the 30%F_u_ sustained for 30 min, the resistance increased about 6% of the original value. Then, definitely, this loading condition is not harmful in terms of the porous structure and even may produce a slight matrix consolidation [3]. However, the material was unable to recover the original parameters when subjected to a higher load and an irreversible damage remains. The loss in the resistance was determined in the order of 7% of the initial value, after a load 0.6F_u_ was applied for 30 min. Moreover, the characteristic time for both recovering periods at unload was about three times shorter than the corresponding characteristic time during load, which points to the faster nature of the time-dependent strain recovery after unloading. 

**Figure 6:**
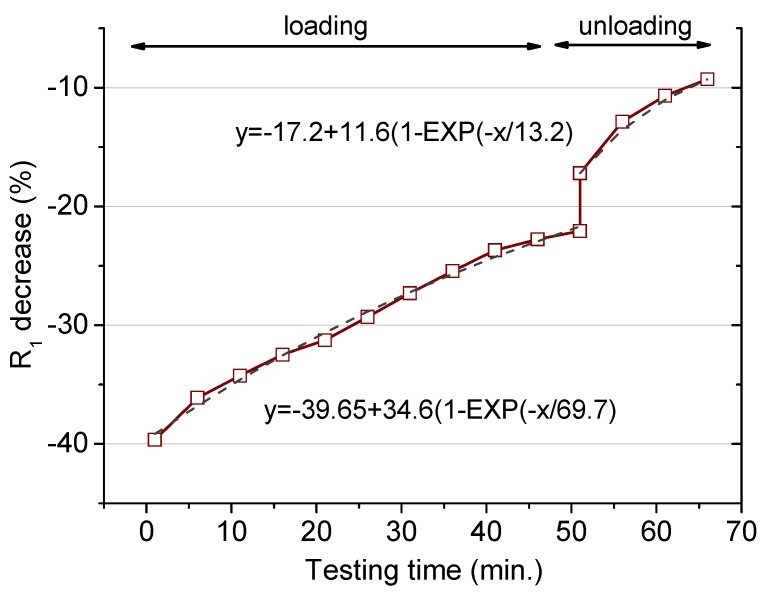
Percentage decrease of the percolating resistance (R_1_ in the model presented in Figure 3) during the cyclic loading period (up to 60 min) and after the load removal (after 60 min). Load applied: 32.9 kN.

A third experiment was performed in order to check the effect of a dynamic load applied for repeated shorter periods. Figure 6 shows the percentage reduction obtained in the R_1_ value during the loading and the unloading stages. The cyclic loading period was defined as 1 loading minute and 4 unloading minutes, this cycle being repeated 10 times. After that, the force was released and, analogously to the previous experiments, the resistance was recorded for an additional 15 min. The trends obtained were similar to those previously indicated, with a first sharp resistance reduction, smooth increase after that, and, finally, after removing the load, sudden rise followed by a less-marked variation. 

The initial decay is in the same order as the value presented in Figure 5 for the same load (39.2 kN), but the further evolution shows a more marked recovery in the resistance for the dynamic test. This observation means that the damage is induced mostly in the first loading step, with no detrimental microcracks formation and/or enlargement in the further stages [22]. The exponential fit points to a less-important matrix deterioration for this particular test. Thus, for a larger number of cycles, the R_1_ reduction would be positioned at around 5% below the initial value. So, under this eventual situation, where a load is repeatedly applied for a short time, the modifications induced at the microstructural level are less relevant in comparison to the case where the same load is constantly applied. The characteristic time obtained from the dynamic loading is 69.7, larger than for the static loading mode. Then, the microstructural modifications under such a cyclic load need a longer period to achieve a steady condition. 

The last part concerning the R_1_ recovery after the load removal shows an analogous variation to that previously explained. A permanent reduction remained after the as–performed test, with a reduction around 5% in the mortar resistance. The characteristic recovery time for the unloading period was in the same order as the times obtained above. An equivalent gradual increase in the resistance, linked to the creep recovery, was obtained independently on the loading conditions. 

### 3.3. Chloride Diffusion Coefficients Assessment

**Figure 7:**
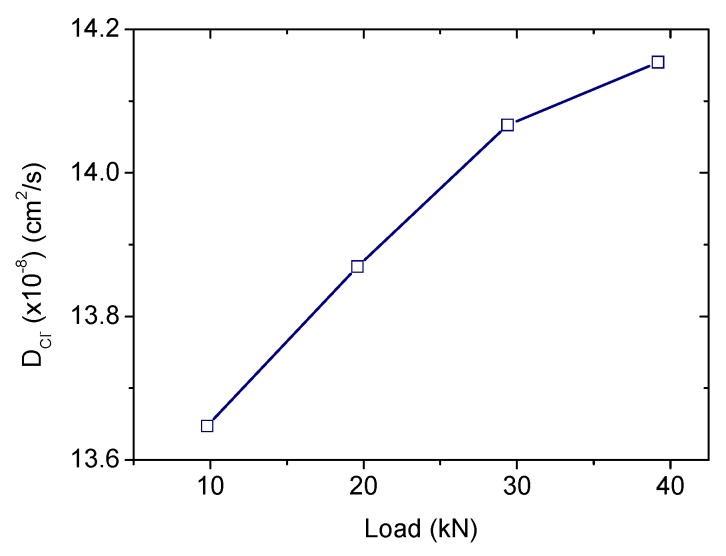
Diffusion coefficient values, obtained from the mortar resistivity, under a punctual sustained load in the range 15%–60%F_u_.

The R_1_ parameter taken from the impedance fits will be used to determine the diffusion coefficients [17]. Concerning the influence of a punctual load, the calculated diffusion coefficients are represented versus the load value in Figure 7. The diffusion coefficient taken from the unloaded specimen is 10.5 × 10^−8^ cm^2^/s, in the same order as those obtained from traditional methods (migration test) in similar samples [17]. The higher the applied load the higher the diffusion coefficient, although the increment is less relevant above 45%F_u_. The diffusion coefficients would be about 30%–35% larger than those obtained in a load-free specimen. Then, a marked effect of an isolated load (usually produced due to traffic, wind, impacts, etc.) in the chloride diffusivity can be confirmed, although no great differences are detected in terms of the particular load value. 

The results obtained from a service load applied for a longer period, that would be the case of structures subjected to a permanent load during their life time, are compiled in Table 5. The values given beyond 30 min were obtained from the exponential fits inserted in Figure 5. For the smaller force, almost no variation was detected after one day of loading, whereas an increase about 20% was obtained for a long-term sustained load of 39.2 kN. These numbers just confirm the results previously discussed, with a marked influence of the loading condition in case it is continually applied. Thus, no detrimental effect was detected in case the load was maintained at the 30%F_u_ but a larger diffusivity was obtained when the load remains at 60%F_u_, in agreement with the predictions provided by other authors [8,33]. A recent publication has revealed the opposite result, concluding a lower diffusivity under longer-term (up to eight weeks) compressive loads below 40%F_u_ [16]. This apparent contradiction is likely related to an enhanced chemical interaction between the chloride ions and the aluminates in the hardened cement phase when a compressive load is sustained for a longer period, as already suggested in this publication [16]. The resulting product of this reaction blocks the capillary pores [24] and finally contributes to the referred diffusivity reduction. In the current research, the testing period could not be prolonged enough to help that reaction [29], and then the possible reduced diffusivity due to it was not compiled. A further study to verify this point is planned. 

The load removal helps to the complete recovery of the original properties in case the load is at 30% of the maximum admissible, even the microstructural changes seem to slightly reduce the chloride diffusion rate. For the highest load, an increased coefficient (~7%) remained after the test was completed. These results, obtained after the load was removed, although they cannot be generalized, give an approach under the studied experimental conditions and reveal differences after the load is released. It is an evidence that the chloride diffusion determined separately, after releasing the load, a procedure followed in many publications in this field, is not representative of the own loading state. Thus, if the chloride diffusivity is measured once the force has been released, the obtained value will be lower than that measured if the load is being applied. 

In case a cyclic load is applied, according to the variations detected in the resistance R_1_, the diffusion coefficient decreases but less markedly in comparison with the same load applied statically for longer, as shown in Table 5. The fit to one day of repeated loading reveals a diffusion coefficient increased by 5%. Then, a permanent damage was also induced with this loading procedure, even after the load was released, with the chloride penetration rate remaining 6% larger than the unloaded specimen. 

## 4. Conclusions

A methodology to determine the chloride diffusion coefficients under the influence of a compressive stress is presented in this study. According to the obtained results, the microstructural modifications detected during the loading tests point to a direct correlation between the chloride diffusivity and the specific loading stage. This study was viable by the incorporation of the impedance technique as the analytical tool, sensitive enough to detect in real time the changes produced under an external load. The main results of this study are summarized as follows:

- The ionic resistance of the studied specimen is influenced by an external compressive load. Since that parameter has been numerically correlated to the chloride diffusivity, an assessment of the diffusion coefficient was provided under the influence of a load.
- In terms of the microstructural modifications, the formation of microcracks was firstly identified followed by a partial matrix reparation. The combined results of the two effects show that a load in the range of 60% of the ultimate strength produces a definitive conductivity increase that was translated into an increase in the chloride diffusion coefficient of 25%. Below that load, the expected modifications are minor, being almost irrelevant for a load of 30% of the ultimate strength.
- A dynamic loading at 60% of the maximum admissible produces a less-important resistance variation, in comparison with the same load sustained permanently. The approximated diffusivity for a repeated number of cycles gives hardly an increment of 5%.

Although further experiments are required in order to delimit the problematic loading range that would definitely affect the chloride diffusivity, this study shows that almost any variable that may affect a loaded concrete structure, such as composition or age, among others, could be easily, rapidly, and realistically assessed by the explained methodology. The results from this investigation evidence the requirement of including an accurate chloride diffusion coefficient to predict the life time of structures under the combined effect of a compressive load and the presence of chlorides. 

## Figures and Tables

**Table 1:** Mechanical and physical properties of the studied mortar specimens.

Flexural Test	Compressive Test	Porosity	Average Pore Diameter
2.9 ± 0.2 kN	66.3 ± 4.9 kN	10.1% ± 0.1%	24.1 ± 5.9 nm

**Table 2:** Summary of the performed experimental tests.

	Experiment #1	Experiment #2	Experiment #3
Load (kN)	9.8 19.6 29.4 39.2	9.8 39.2	39.2/unloading
Period	10 sec	30 min	1 min/4 min

**Table 3:** Fitting parameters of the spectra introduced in Figure 2, compiling the influence of a punctual increasing force.

	R_1_ (kΩ.cm^2^)	C_1_ (pF/cm^2^)	R_2_ (kΩ.cm^2^)	C_2_ (pF/cm^2^)	δ (nm)
no load	6.19	2.36	92.25	4.00	19.2
9.8 kN	4.76	2.54	62.41	5.29	18.2
19.6 kN	4.68	2.49	64.24	4.87	17.7
29.4 kN	4.61	2.48	61.39	4.98	17.2
39.2 kN	4.59	2.44	60.06	5.24	17.7

**Table 4:** Fitting parameters of the spectra introduced in Figure 4, compiling the influence of the loading time under 30%F_u_ and 60%F_u_.

	R_1_ (kΩ.cm^2^)		C_1_ (pF/cm^2^)		δ (nm)	
	19.6 kN	39.2 kN	19.6 kN	39.2 kN	19.6 kN	39.2 kN
no load	6.7	10.3	2.2	1.9	18.3	21.2
initial	4.3	6.4	2.6	2.1	23	14
30 min	5.4	7.2	2.5	2.2	21.1	16

**Table 5:** Diffusion coefficient values, obtained from the mortar resistivity, after loading at 30% and 60%F_u_ for 30 min and under a dynamic load at 60%F_u_.

	19.6 kN	39.2 kN
unloaded	10.0 × 10^−8^ cm^2^/s	6.3 × 10^−8^ cm^2^/s
30 min	12.1 × 10^−8^ cm^2^/s	8.9 × 10^−8^ cm^2^/s
1 day (fit)	10.2 × 10^−8^ cm^2^/s	7.9 × 10^−8^ cm^2^/s
load removal—1 day (fit)	9.4 × 10^−8^ cm^2^/s	6.8 × 10^−8^ cm^2^/s
Cyclic loading		
unloaded	-	10.1 × 10^−8^ cm^2^/s
30 min	-	13.0 × 10^−8^ cm^2^/s
1 day (fit)	-	10.6 × 10^−8^ cm^2^/s
load removal—1 day (fit)	-	10.7 × 10^−8^ cm^2^/s
